# Longer Arousal, Better Semen: Effects of Extended Pre-Ejaculatory Arousal on Semen Parameters in Infertile Men

**DOI:** 10.3390/diagnostics15243186

**Published:** 2025-12-13

**Authors:** Murat Beyatlı, Hasan Samet Güngör, Tuncel Uzel, Resul Sobay, Abdurrahman İnkaya, Mehmet Umut Evci, Rıdvan Özbek, Ahmet Tahra, Eyüp Veli Küçük

**Affiliations:** 1 Department of Urology, Umraniye Training and Research Hospital, Istanbul 34093, Türkiye; drsametgngr@gmail.com (H.S.G.); drresulsobay@gmail.com (R.S.); ainkaya@hotmail.com (A.İ.); umutevci.md@gmail.com (M.U.E.); ahmettahra@gmail.com (A.T.); eyupveli@gmail.com (E.V.K.); 2 Department of Urology, University of Health Sciences Dr. Abdurrahman Yurtaslan Ankara Oncology Research and Training Hospital, Ankara 06200, Türkiye; tunceluzelmd@gmail.com; 3 Department of Urology, Kastamonu University, Kastamonu 37150, Türkiye; rdvnzbk@yandex.com

**Keywords:** assisted reproduction, erotic video, male infertility, motility, semen parameters, sexual arousal, urology

## Abstract

**Background/Objectives**: To examine whether extending sexual arousal through lengthened erotic video viewing during semen collection improves semen parameters in infertile men. **Methods**: In this prospective within-subject study, 205 infertile men supplied semen samples in two sessions about four weeks apart and followed strict uniform protocols. In the first session, participants ejaculated after the customary viewing length of erotic videos. During the second session, viewing time was increased prior to ejaculation. Semen volume, sperm concentration, total motility, progressive motility, and morphology were assessed as per the WHO 2021 guidelines. Comparisons were analyzed using paired samples *t*-test or Wilcoxon signed-rank test and correlation analysis was performed using Spearman’s rank correlation. **Results**: Median duration of erotic video watching increased from 8 min (IQR: 5–10) to 15 min (IQR: 11–20) (*p* < 0.001). Moreover, extended arousal was associated with increased sperm concentration (from 19 × 10^6^/mL to 24 × 10^6^/mL, *p* < 0.001), total motility (43% to 46%, *p* < 0.001), and progressive motility (33% to 37%, *p* < 0.001) with unchanged morphology (*p* = 0.053). Increases in arousal duration exhibited a weak but significant correlation with changes in concentration (ρ = 0.21, *p* = 0.003), total motility (ρ = 0.27, *p* < 0.001), and progressive motility (ρ = 0.22, *p* = 0.002). Subgroup analysis showed improvements in non-smokers but not in smokers. **Conclusions**: Extended erotic stimulation during semen sample collection significantly enhances sperm motility and modestly increases concentration in men with infertility, albeit without changing morphology. These findings suggest that extended erotic stimulation during semen collection may be a simple, non-invasive strategy to help optimize semen quality in infertile men, especially non-smokers.

## 1. Introduction

Male infertility continues to emerge as the ultimate health concern; hence, it accounts for about half of all infertility cases among couples worldwide [1]. Semen analysis remains the cornerstone of male infertility evaluation, giving valuable information on sperm concentration, motility, morphology, and ejaculate volume [2,3]. Semen parameters, however, can be affected by several intrinsic and extrinsic factors, including age and lifestyle habits, duration of abstinence, environmental exposures, and the state of arousal, from a psychological or sexual perspective, while providing the sample [4,5].

There had been previous investigations on the effect of sexual stimulation on semen quality, mostly through sexually explicit visual stimuli [6]. In a landmark experiment, Yamamoto et al. illustrated that masturbation with visual images (VIM) significantly improved the volume, motility, and functional parameters of sperm both in normozoospermic and cryptozoospermic men, relative to masturbation without VIM [7]. Intriguingly, for cryptozoospermic males, the application of VIM sometimes ensued in the detection of motile spermatozoa, thus sometimes circumventing the need for invasive testicular biopsy [7]. Considering the above findings, enhanced sexual stimulation may augment prostatic and seminal vesicular secretions through increased sympathetic nervous system activity, potentially optimizing the biochemical microenvironment and improving sperm functional parameters in the ejaculate.

Recent studies have been dealing with other alterable parameters during semen collection. Sertkaya et al. studied semen quality under sexual arousal during a fixed 3-day abstinence period in infertile men and found that sperm parameters could be suitably influenced by the timing of ejaculation [8]. Likewise, Rosenkjær et al. examined variables related to collection in assisted reproductive technology (ART) settings, emphasizing optimization in semen collection protocol to ensure a standard and quality sample [9]. Nevertheless, the effect of lengthening sexual stimulation has been somewhat less considered, at least so far, especially for infertile men.

Hence, this study was designed to close this knowledge gap by assessing whether an increase in sexual arousal by extended exposure to sexually explicit films during semen collection will improve semen parameters in infertile men. Previous studies have mainly compared situations in settings with the presence versus absence of sexual stimuli, but our study contrasts two conditions under controlled circumstances within the same individuals: standard (short) viewing and lengthened viewing. The observation pertaining to infertile men may carry direct clinical value in terms of optimizing semen quality in a population for whom every sample is critical to diagnosis and treatment.

## 2. Materials and Methods

This prospective study was conducted between November 2024 and January 2025 in male patients presenting to our clinic with infertility. The sample size was calculated using G*Power software (version 3.1.9.2; Heinrich Heine University, Düsseldorf, Germany). Assuming a standard effect size of 0.20, a two-tailed α level of 0.05, and a desired statistical power (1 − β) of 0.80, the required sample size was determined to be 197. To account for an anticipated 20% loss, approximately 240 patients were planned for inclusion. The target sample size was met, with 205 participants included in the final analysis.

Inclusion criteria were as follows: (i) men aged 18–45 years; (ii) a diagnosis of infertility, defined as failure to conceive after at least 12 months of regular unprotected intercourse; (iii) ability to provide two semen samples under the study conditions approximately four weeks apart; and (iv) provision of written informed consent.

Arousal was assessed solely based on objective measurement of viewing duration using digital timers, without employing validated self-reported arousal questionnaires. Obstructive azoospermia, severe azoospermia/oligozoospermia, abnormal hormone parameters, recent testicular surgery/urological investigation within six months, genital infection within ninety days, consumption of drugs that halt spermatogenesis, and disease that depresses semen quality were exclusion criteria. Smoking was self-reported, and participants were categorized as smokers and non-smokers. Out of 240 individuals screened, 35 were not put on further analysis (5 obstructive azoospermia, 7 severe oligozoospermia/azoospermia, 4 abnormal hormone parameters, 6 recent testicular surgery, 5 recent genital infection, 4 medicine, and 4 disease), leaving 205 volunteers for final analysis.

Semen collection was performed in private, standardized rooms specifically designed to optimize patient comfort and minimize psychological stress that could adversely affect arousal and semen quality. Each collection room was equipped with identical amenities including comfortable seating, ambient lighting, temperature control maintained at 22–24 °C, soundproofing for privacy, and high-definition audiovisual equipment. The rooms were designed with calming décor and ergonomic features to create a relaxed environment. Participants were assured complete privacy with ‘do not disturb’ signage and were given unlimited time for the collection process without time constraints to reduce performance anxiety. Staff members were trained to minimize interactions and provide clear, supportive instructions to further reduce psychological stress. Participants were instructed to start the selected video when beginning sexual stimulation and to continue viewing until ejaculation. Pre-ejaculatory arousal time was operationalized as the viewing duration (in minutes) from video onset to ejaculation, and this duration was recorded for each semen collection.

Video content was partially standardized using a curated library of erotic videos. The library consisted of professionally produced clips with comparable duration and explicitness, predominantly heterosexual content, and without extreme or violent material. Content categories were limited and partially standardized to reduce excessive variability in arousal triggers, while still offering sufficient diversity to accommodate individual preferences and maintain consistency in production quality and content appropriateness. Participants were allowed to select preferred content from within this standardized library to optimize individual arousal quality, as personal preferences significantly influence arousal response. While this approach may introduce some variability in stimulus content, it reflects real-world clinical practice and ensures adequate arousals for all participants. All video selections were documented, and no significant differences in arousal duration were observed between different content categories.

Infertility was defined as an inability to conceive after 12 months of regular unprotected intercourse.

Participants gave two semen samples about four weeks apart under identical environmental circumstances and after 3–5 days of abstinence in response to their typical pre-ejaculatory protocol. During the approximately 4-week interval between collections, lifestyle habits (e.g., diet, physical activity), medication use, and other environmental factors were not systematically controlled or recorded. The first sample (Film Duration 1) was collected after the participant’s typical pre-ejaculatory schedule, whereas the second sample (Film Duration 2) was taken after actively extended sexual excitation under the same circumstances. Semen analysis was conducted by the same seasoned andrology technician according to World Health Organization 2021 criteria [10], including measurements of volume (mL), sperm concentration (million/mL), total and progressive motility (%), and morphology (%) after liquefaction at 37 °C for up to 60 min. Total sperm count was calculated by multiplying semen volume (mL) by sperm concentration (million/mL).

To minimize analytical bias, the experienced andrology technician performing all semen analyses was blinded to the sample collection order and the specific arousal protocol used for each specimen. Samples were coded using participant identification numbers and collection dates only, without indicating whether they represented the first (standard arousal) or second (extended arousal) sample. The technician was not informed about the study objectives or expected outcomes until after all analyses were completed. This blinding protocol ensured objective assessment of semen parameters according to WHO 2021 guidelines without potential bias influencing the analytical results.

We aimed to investigate how extended sexual arousal would affect semen quality in the same individuals. Principal outcomes were the changes in semen volume, sperm count, total motility, progressive motility, and morphology differences between baseline (standard arousal) and follow-up (extended arousal) samples. Secondary outcomes were changes in percentages of film duration from one session to another, correlations between percentages of change in film duration and changes in semen measures, and subgroup analysis based on smoking status.

### Statistical Analysis

The distribution of continuous measures was ascertained from the Kolmogorov–Smirnov test. Normally distributed measures were expressed as mean ± standard deviation (SD) and compared across sessions through a paired-samples *t*-test. Non-normally distributed measures were expressed as median (interquartile range, IQR) and compared using the Wilcoxon signed-rank test. Categorical measures were expressed as counts (percentages) and compared through McNemar’s test when applicable.

Correlations between the percentage change in viewing duration and changes in semen parameters were determined with Spearman’s rank correlation coefficient. Subgroup analysis based on smoking status deployed this same paired approach within subgroups. All statistical procedures were performed with SPSS version 22.0 (IBM Corp., Armonk, NY, USA). A *p*-value of <0.05 was considered statistically significant.

## 3. Results

A total of 205 infertile men were included in the final analysis, with a median age of 32 years (IQR: 29–36) and a median BMI of 27 kg/m^2^ (IQR: 24–28). Among these, 87 participants (42.4%) were smokers. The median sexual abstinence period was identical for both semen collections (4 days, IQR: 3–4). Median arousal time increased from 8 min (IQR: 5–10) during the first session to 15 min (IQR: 11–20) in the second session, corresponding to a median increase of 7 min (IQR: 5–10) (*p* < 0.001, Wilcoxon signed-rank test). The median percentage increase in arousal duration was 87.5%.

**Figure 1:**
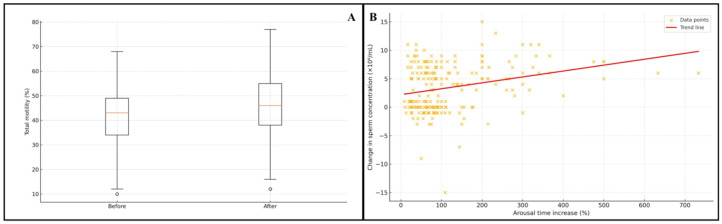
(**A**) Change in total motility before and after extended arousal (box plot). (**B**) Association between percentage increase in arousal time and change in sperm concentration (scatter plot).

Lengthening pre-ejaculatory arousal was associated with statistically significant improvements in several semen parameters (Table 1). Sperm concentration increased from a median of 19 × 10^6^/mL (IQR: 12–41) to 24 × 10^6^/mL (IQR: 14–48) (*p* < 0.001, Wilcoxon signed-rank test). Total motility improved from a median of 43% (IQR: 34–49.5) to 46% (IQR: 38–55) (*p* < 0.001), and progressive motility increased from 33% (IQR: 26–42) to 37% (IQR: 28–46.5) (*p* < 0.001) (Figure 1). Morphology did not change significantly (*p* = 0.053).

Spearman correlation analysis revealed weak but statistically significant positive associations between the magnitude of arousal time increase and changes in sperm concentration (ρ = 0.209, *p* = 0.003), total motility (ρ = 0.272, *p* < 0.001), and progressive motility (ρ = 0.218, *p* = 0.002). No association was observed with morphology (ρ = −0.030, *p* = 0.667) (Table 2) (Figure 1).

In non-smokers (*n* = 118), sperm concentration, total motility, and progressive motility significantly increased from first to second sample (all *p* < 0.001), but semen volume and morphology were unaltered. For smokers (*n* = 87), no differences were seen in semen parameters despite a significant increase in duration of arousal (*p* < 0.001) (Table 3).

Exploratory subgroup analyses stratified by age (<35 vs. ≥35 years), BMI (<25 vs. ≥25 kg/m^2^), and baseline total motility (<40% vs. ≥40%) did not reveal any consistent statistically significant differences in the magnitude of improvement in semen parameters between strata (all *p* > 0.05).

Total sperm count, calculated as semen volume multiplied by sperm concentration, increased significantly from a median of 56.0 × 10^6^ (IQR: 36.0–114.0) in the first sample to 70.0 × 10^6^ (IQR: 42.0–132.0) in the second sample (*p* < 0.001, Wilcoxon signed-rank test). Total motile sperm count, representing the number of motile spermatozoa available for fertilization, improved from a median of 22.5 × 10^6^ (IQR: 14.2–48.6) to 29.1 × 10^6^ (IQR: 17.8–58.8) (*p* < 0.001).

Clinical significance analysis based on WHO 2021 reference values revealed meaningful improvements in subfertile participants. Among men with baseline sperm concentration ≤15 × 10^6^/mL, 27 participants (13.2%) achieved concentrations >15 × 10^6^/mL after extended arousal. Similarly, 27 men (13.2%) with baseline total motility ≤40% improved to >40%, and 26 participants (12.7%) with baseline progressive motility ≤32% achieved >32% progressive motility in the second sample.

## 4. Discussion

This study is, to our knowledge, the first to specifically manipulate the duration of pre-ejaculatory erotic video viewing in an exclusively infertile cohort using a within-subject design. We found that extended viewing time resulted in statistically significant improvements in total and progressive motility, as well as sperm concentration, whereas morphology remained unchanged. Viewing duration showed a weak but statistically significant correlation with motility improvement. Among smokers, although this duration increased significantly, no enhancement in semen parameters was observed. These findings have implications for optimizing semen collection protocols in assisted reproduction, particularly in male factor infertility.

Total and progressive motilities were significantly enhanced when subjects were exposed to erotic content for an extensive duration prior to collection. This corroborates with Yamamoto et al., who showed that VIM enhanced quantitative and qualitative motility measures in normozoospermic and cryptozoospermic subjects [7]. Sertkaya et al. similarly showed an effect in an admixed sample of fertile and infertile subjects [8]. In this study, an effect was seen despite subjects being uniformly infertile, a category that, in general, would have more impaired baseline motilities [11].

The magnitude of improvement in our infertile cohort was smaller than in Yamamoto’s normozoospermic group [7], suggesting that while the physiological mechanisms underlying enhanced motility, such as increased prostatic secretory function, improved seminal plasma composition, and optimized emission from the vas deferens, are likely preserved, their effect size may be attenuated in men with spermatogenic or accessory gland dysfunction. Chronic oxidative stress or structural flagellar defects, which are more prevalent in infertile men, may further limit the potential benefit of extended arousal [12].

In contrast, sperm morphology did not change significantly following extended stimulation. This differs from Yamamoto et al., who reported modest improvements in normozoospermic men [7], and from Sertkaya et al., who noted minor changes [8]. The absence of such an effect in our population may be due to the fact that morphological abnormalities typically reflect long-term spermatogenic insults (e.g., varicocele, genetic defects, chronic inflammation) rather than acute factors present at the time of ejaculation. Therefore, alterations in the ejaculatory environment, including accessory gland secretion or seminal pH, are unlikely to affect morphology in the short term [13,14]. Furthermore, because many participants had severe teratozoospermia, the baseline proportion of normal forms was low, potentially creating a floor effect that limited the detection of subtle morphological changes.

We detected a statistically significant, although weak, positive correlation between duration of erotic film viewing and enhanced motility of the sperm. This is consistent with the model proposed by Yamamoto et al., who suggested that increased sexual stimulation produces increased prostatic and seminal vesicular secretions, which improve the biochemical environment for motility of the sperm [7]. Nonetheless, the fact that our correlation was weak suggests that additional factors, including inter-individual variability of arousal response, psychogenic modulation, and baseline accessory gland function, probably regulate this relationship.

While the observed improvements were statistically significant, the effect sizes were modest, requiring careful interpretation of clinical significance. The median increase in total motile sperm count from 22.5 to 29.1 million represents a potentially meaningful improvement, as the literature suggests total motile sperm counts >10–15 million are associated with improved intrauterine insemination (IUI) success rates. The 4% median improvement in progressive motility may enhance fertilization potential in both IUI and in vitro fertilization (IVF) cycles, as progressive motility >32% is associated with better clinical outcomes. However, the correlation coefficients of 0.21–0.27 indicate that extended arousal explains only 4–7% of the variance in semen parameter improvements, suggesting modest clinical impact at the population level.

The effect sizes observed in our study were modest, with correlation coefficients ranging from 0.21 to 0.27, indicating weak to moderate associations between arousal duration and semen parameter improvements. While these correlations were statistically significant, they suggest that extended arousal explains only a small proportion of the variance in semen quality improvements. These modest effect sizes reflect the multifactorial nature of semen quality and indicate that extended arousal represents an optimization tool rather than a transformative intervention for male infertility. Clinicians should interpret these findings within the context of comprehensive male fertility evaluation and management.

Sertkaya et al. did not exactly measure the correlation between duration of stimulation and changes in semen; they just categorized exposure as “with” or “without” erotic stimulus [8]. Our quantitative method exemplifies that longer exposure can provide incremental motility advantages, yet their size declines after a specific duration, implying a physiological ceiling effect.

In our study, extended exposure to erotic films significantly increased sperm concentration, total motility, and progressive motility in non-smokers, but no noticeable increments were observed in smokers, even with longer exposure times. Smoking is a known adverse determinant of semen quality, mostly through mechanisms related to oxidative stress, DNA lesions, and impaired accessory gland function [15,16]. Our data imply that these chronic, harmful impacts may put a cap on the acute effect of prolonged erotic stimulation in habitual smokers, possibly secondary to structural and functional deficits in spermatogenesis and seminal plasma composition.

The absence of improvements in smoking participants warrants acknowledgment of potential statistical power limitations. With 87 smokers in our cohort, our study may have been underpowered to detect modest improvements in this subgroup. The chronic oxidative stress and DNA damage associated with smoking may attenuate the acute benefits of extended arousal, creating a higher threshold for detectable improvement. Future studies with larger sample sizes specifically designed to evaluate interventions in smoking infertile men are needed to definitively determine whether extended arousal protocols can benefit this population.

Clinically, this research implies that erotic stimulation protocols can be utilized in smokers within ART, even though their overall outcome is worse than that of non-smokers. Use of prolonged erotic stimulation before semen collection is an easy, non-invasive method to optimize motility and concentration in infertile men, especially when baseline motility is suboptimal for IUI or when higher motility fractions are needed for intracytoplasmic sperm injection (ICSI) [17]. The absence of morphologic change verifies that this intervention is an acute optimization measure, not a therapy for underlying intrinsic spermatogenic defects.

The weak correlation between duration of viewing and motility increments also implies that excessively long stimulation is not required; practical protocols must strike a balance between patient comfort and optimal semen quality.

The mechanisms underlying the observed improvements likely involve multiple interconnected pathways. Psychogenic arousal enhances sympathetic nervous system activity, potentially optimizing the coordinated contractions of the vas deferens, seminal vesicles, and prostate during ejaculation, leading to more complete emission of higher-quality spermatozoa [18]. Extended arousal may also modulate the hormonal milieu, including increased testosterone, oxytocin, and prolactin levels, which can influence sperm function and accessory gland secretions [19,20]. Neurophysiological arousal may reduce oxidative stress through enhanced antioxidant enzyme activity and improved cellular energy metabolism, protecting sperm from DNA damage and membrane lipid peroxidation [15]. Additionally, prolonged stimulation may allow for more thorough mixing of sperm with seminal plasma components, optimizing pH, osmolality, and nutrient availability [21]. Psychological factors including reduced performance anxiety, enhanced sexual satisfaction, and improved confidence may also contribute through neuroendocrine pathways affecting the hypothalamic-pituitary-gonadal axis [22].

Several limitations should be acknowledged. First, we acknowledge that our study is limited to short-term laboratory outcomes and does not provide data on fertilization rates, clinical pregnancy rates, or live birth rates. While we observed statistically significant improvements in sperm motility and concentration, the clinical significance of these changes requires validation through outcome-based studies. Future research should investigate whether the modest improvements in semen parameters translate to enhanced fertilization rates in IVF cycles or improved pregnancy rates in IUI. Studies examining the correlation between extended arousal protocols and actual reproductive outcomes in assisted reproductive technology cycles would provide definitive evidence of clinical utility. We did not formally assess psychological factors such as anxiety levels, stress, or sexual satisfaction using validated questionnaires, which could have provided insights into the mechanisms underlying our observations. Additionally, we did not measure biochemical correlates of accessory gland function (such as citrate, zinc, or fructose levels) or hormonal parameters (testosterone, prolactin, oxytocin) that might explain the improvements in semen parameters. The absence of these measurements limits our understanding of the physiological mechanisms responsible for the observed changes. Although our sample size was sufficient for primary outcomes, it may have limited power for subgroup analyses, including the smoker versus non-smoker comparison. This study was conducted in a single fertility center, which may limit the generalizability of our findings to different clinical settings, populations, or cultural contexts. Variations in collection room environments, video equipment quality, privacy levels, and patient demographics across different centers could influence the reproducibility of our results. Despite partial standardization of the erotic video library, residual differences in content type and intensity may still have influenced individual arousal responses and represent an additional source of variability. Multi-center studies involving diverse populations and standardized protocols would strengthen the evidence base and enhance clinical practice applicability of extended arousal protocols. A significant limitation of our study design is the absence of crossover randomization and counterbalancing. Another important limitation is that sexual arousal was operationalized solely as viewing duration of erotic material, without concurrent physiological measures (e.g., heart rate, skin conductance) or standardized psychometric scales to assess subjective arousal.

Future studies should integrate multidimensional assessment of sexual arousal, combining behavioral, physiological, and validated psychological measures, and should also examine structured erotic stimulation protocols in larger, more diverse cohorts, ideally using crossover or counterbalanced designs. Comparative studies including both infertile and fertile men, and protocols allowing individualized material selection, could further clarify how underlying pathology and stimulus characteristics influence responsiveness to stimulation and semen quality.

## 5. Conclusions

This study fills a key gap with a quantitative assessment of the effect of extended erotic stimulation on semen quality exclusively in infertile men, using a within-subject design to minimize inter-individual differences. We demonstrated that moderate increments of exposure time are able to enhance motility and concentration, particularly in non-smokers, in a simple, non-invasive manner to optimize collection regimes in assisted reproduction, but display limited reactivity of sperm morphology and decreased effectiveness in smokers. Further research needs to be conducted to explore the broader clinical effects of this intervention.

It is crucial to emphasize that extended erotic stimulation represents an optimization tool rather than a treatment for underlying spermatogenic dysfunction or male infertility. This intervention does not address fundamental causes of poor semen quality such as varicocele, hormonal imbalances, genetic defects, or infections, but rather maximizes the functional potential of existing sperm production. Extended arousal protocols should be considered as an adjunctive optimization strategy within comprehensive male fertility evaluation and management, not as a standalone therapeutic intervention. Clinicians should continue to pursue appropriate diagnostic workup and evidence-based treatments for identified causes of male infertility while potentially incorporating extended arousal protocols to optimize semen collection for ART procedures.

## Figures and Tables

**Table 1:** Comparison of the first and second semen analyses of 205 participants.

Parameter	First Semen Analysis Median (IQR)	Second Semen Analysis Median (IQR)	*p*-Value *
Abstinence period (days)	4 (3–4)	4 (3–4)	-
Semen volume (mL)	3 (2–4)	3 (2–4)	0.317
Sperm concentration (10^6^/mL)	19 (12–41)	24 (14–48)	**<0.001**
Total motility (PR + NP, %)	43 (34–49.5)	46 (38–55)	**<0.001**
Progressive motility (PR, %)	33 (26–42)	37 (28–46.5)	**<0.001**
Sperm morphology (%)	3 (2–5)	3 (2–4)	0.053
Duration of sexual arousal (minutes)	8 (5–10)	15 (11–20)	**<0.001**

IQR: interquartile range, PR: progressive, NP: non-progressive. * Wilcoxon signed-rank test. Significant *p*-values are written in bold.

**Table 2:** Spearman correlations between the percentage increase in arousal time and changes in semen parameters.

Change Variable	Spearman’s ρ	*p*-Value
Sperm concentration	0.209	**0.003**
Total motility	0.272	**<0.001**
Progressive motility	0.218	**0.002**
Morphology	−0.030	0.667

Significant *p*-values are written in bold.

**Table 3:** Comparison of first and second semen analyses among non-smoking patients (*n* = 118) and smoking patients (*n* = 87).

	Non-Smoking Patients (*n* = 118)			Smoking Patients (*n* = 87)		
	First Semen Analysis Median (IQR)	Second Semen Analysis Median (IQR)	*p*-Value *	First Semen Analysis Median (IQR)	Second Semen Analysis Median (IQR)	*p*-Value *
Abstinence period (days)	4 (3–4)	4 (3–4)	-	4 (3–4)	4 (3–4)	-
Semen volume (mL)	3 (2–4)	3 (2–4)	-	2 (2–4)	2 (2–4)	0.317
Sperm concentration (10^6^/mL)	21.5 (12–45)	28 (17–53)	**<0.001**	19 (12–38)	19 (12–38)	0.322
Total motility (PR + NP, %)	42 (34.75–51)	50 (42.75–59)	**<0.001**	43 (33–49)	43 (34–50)	0.686
Progressive motility (PR, %)	33 (27–42)	39 (33–48)	**<0.001**	35 (24–42)	34 (25–42)	0.147
Sperm morphology (%)	3 (2–5)	3 (2–4.25)	0.223	3 (2–5)	3 (2–5)	0.123
Duration of sexual arousal (minutes)	6 (4–7)	13 (9–18)	**<0.001**	10 (9–11)	18 (14–21)	**<0.001**

IQR: interquartile range, PR: progressive, NP: non-progressive. * Wilcoxon signed-rank test. Significant *p*-values are written in bold.

## Data Availability

The data presented in this study are available on request from the corresponding author due to privacy.

## Abbreviations

The following abbreviations are used in this manuscript:

ART	Assisted reproductive technology
BMI	Body mass index
DNA	Deoxyribonucleic acid
ICSI	Intracytoplasmic sperm injection
IQR	Interquartile range
IUI	Intrauterine insemination
IVF	In vitro fertilization
NP	Non-progressive (motility)
PR	Progressive (motility)
VIM	Visual images for masturbation
WHO	World Health Organization
